# Association between diabetes mellitus and tinnitus: A meta-analysis

**DOI:** 10.17305/bb.2024.11634

**Published:** 2025-01-20

**Authors:** Shi Luo, Jianxue Wen, Qilong Bao, Haibo Ou, Shuting Yi, Peng Peng

**Affiliations:** 1 Department of Otorhinolaryngology and Head and Neck Surgery, The Affiliated Zhuzhou Hospital Xiangya Medical College CSU, Zhuzhou, China; 2 Department of Otorhinolaryngology and Head and Neck Surgery, Xiangya Second Hospital of Central South University, Changsha, China

**Keywords:** Tinnitus, diabetes mellitus, DM, prevalence, risk factor, meta-analysis

## Abstract

Diabetes mellitus (DM) has been suggested as a potential risk factor for tinnitus, but evidence remains inconclusive. This meta-analysis aimed to evaluate the association between DM and tinnitus by systematically reviewing and synthesizing data from observational studies. A comprehensive literature search was conducted in PubMed, Embase, and Web of Science up to August 16, 2024. Observational studies with a sample size of at least 100 participants that assessed the relationship between DM and tinnitus were included. Studies involving populations with specific diseases were excluded. Odds ratios (ORs) and 95% confidence intervals (CIs) were pooled using a random-effects model. Study quality was assessed using the Newcastle-Ottawa Scale (NOS), and sensitivity and subgroup analyses were performed. Publication bias was evaluated using funnel plots and Egger’s regression test. Twelve studies comprising 2,277,719 participants were included. The pooled analysis revealed a significant association between DM and tinnitus (OR: 1.18, 95% CI: 1.06–1.31, *P* ═ 0.002), with moderate heterogeneity (*I*^2^ ═ 51%). Sensitivity analyses confirmed the robustness of these findings. Subgroup analyses showed no significant differences by geographical region, mean age, sex distribution, tinnitus diagnosis method, or model used for association estimation. Publication bias was not detected (Egger’s test *P* ═ 0.29). These findings suggest that DM is significantly associated with an increased risk of tinnitus. Further research is warranted to explore underlying mechanisms and causal relationships. Nonetheless, the results underscore the importance of monitoring tinnitus in patients with diabetes.

## Introduction

Tinnitus, the perception of sound without an external source, is a common auditory condition affecting a significant portion of the global population [1, 2]. Epidemiological studies estimate that 10%–15% of adults experience chronic tinnitus, with prevalence rising with age and reaching up to 30% in elderly individuals [1, 3]. The condition can severely impair quality of life, causing disturbances in sleep, concentration, emotional well-being, and even leading to mental health issues, such as anxiety and depression [4, 5]. While some individuals adapt to tinnitus, others suffer from a persistent, debilitating form that disrupts daily functioning [6, 7]. Given its widespread prevalence and potential severity, understanding the risk factors for tinnitus is critical for developing effective prevention and management strategies. The etiology of tinnitus is multifactorial, involving a complex interplay of genetic, environmental, and medical factors [8, 9]. Established risk factors include hearing loss, noise exposure, ototoxic medications, head and neck trauma, and psychological stress [10]. Additionally, metabolic and cardiovascular conditions, such as hypertension and dyslipidemia, have been implicated [11]. However, these factors alone do not fully account for the variability in tinnitus prevalence and severity, underscoring the need to identify additional modifiable risk factors. One emerging area of interest is the role of systemic conditions, such as diabetes mellitus (DM), in the pathophysiology of tinnitus [12]. DM, a chronic metabolic disorder characterized by hyperglycemia, is associated with various microvascular and neural complications, raising questions about its potential impact on auditory dysfunction, including tinnitus [13]. The biological mechanisms linking DM to tinnitus remain unclear, but several pathways have been proposed. Hyperglycemia can induce oxidative stress and inflammation, which may impair cochlear function and damage auditory pathways [14]. Microvascular damage—a hallmark of DM—could reduce blood flow to auditory structures, further contributing to tinnitus [12]. Neuropathy, another common DM complication, may affect the auditory nerve, resulting in sensory dysfunction. Additionally, insulin resistance and glucose dysregulation might alter neurotransmitter activity, exacerbating tinnitus symptoms [13, 15]. Despite these plausible mechanisms, the relationship between DM and tinnitus remains under-researched, with inconsistent findings from existing studies [16–27]. While some studies report a significant association between DM and tinnitus [17, 18, 20, 21, 23], others find no correlation between DM and auditory function [16, 19, 22, 24–27]. These discrepancies highlight the need for a comprehensive evaluation of the evidence. To address this gap, we conducted a meta-analysis to synthesize current findings, assess the strength of the association between DM and tinnitus, and explore potential sources of heterogeneity across studies. By summarizing observational study results, this analysis aims to enhance our understanding of tinnitus etiology and guide future research and clinical practices in this area.

## Materials and methods

This meta-analysis was conducted following the Meta-analysis Of Observational Studies in Epidemiology (MOOSE) guidelines [28] and the Cochrane Handbook for Systematic Reviews and Meta-analysis [29]. The study protocol has been registered in PROSPERO under the identifier CRD42024594905.

### Literature search

A comprehensive literature search was conducted across the PubMed, Embase, and Web of Science databases to identify studies published up to August 16, 2024, that evaluated the association between DM and tinnitus. The search strategy utilized terms related to “diabetes,” “diabetes mellitus,” and “tinnitus.” Detailed search strategies for each database are provided in Supplemental data. Only studies published in peer-reviewed journals as full-length articles in English or Chinese were included. Additionally, as part of the manual screening process, the references of relevant original and review articles were reviewed to identify potential additional studies.

### Inclusion and exclusion criteria

Eligible studies met the following inclusion criteria according to the PICOS principle were included.

Population (P): Studies including individuals with and without DM.

Intervention/Exposure (I): Presence of DM.

Comparison (C): Absence of DM.

Outcome (O): Reported association between DM and tinnitus, with sufficient data to calculate odds ratios (ORs) and 95% confidence intervals (CIs).

Study design (S): Observational studies (cohort, case-control, or cross-sectional) with a minimum sample size of 100 participants. We included studies with a minimum sample size of 100 participants to enhance the reliability of our findings. Smaller studies are more prone to variability and biases, such as publication bias and confounding effects, which could undermine the robustness of pooled estimates [30]. Evidence from meta-analytic methodology indicates that underpowered or small-sample studies may inflate effect sizes or lead to imprecise conclusions [30].

Studies involving populations with specific diseases (e.g., cardiovascular, neurological, or renal diseases) were excluded to minimize confounding effects. Additionally, reviews, case reports, editorials, and animal studies were not included. For overlapping patient populations, the study with the largest sample size was chosen for inclusion in the meta-analysis.

### Data extraction

Two independent reviewers screened the titles, abstracts, and full texts of the studies and extracted relevant data using a standardized data extraction form. Discrepancies were resolved through discussion or consultation with a third reviewer. Extracted data included study characteristics (e.g., author, year of publication, country, and study design), participant characteristics (e.g., source of the population, sample size, age, and gender distribution), methods for diagnosing DM, type of DM, number of patients with DM, methods for validating tinnitus cases, number of patients with tinnitus, and confounders adjusted for when estimating the association between DM and tinnitus. If multiple effect estimates were reported, the most fully adjusted model was extracted.

### Quality assessment

The quality of the included studies was assessed using the Newcastle-Ottawa Scale (NOS), which evaluates three domains: the selection of study groups, the comparability of groups, and the ascertainment of outcomes [31]. Each study received a score ranging from 0 to 9 stars, with studies scoring 6 stars or more classified as moderate-to-high quality. The risk of bias was independently evaluated by two reviewers, with disagreements resolved through discussion. Studies identified as having a high risk of bias were further analyzed through sensitivity analysis.

### Statistical analysis

OR with 95% CI were calculated to examine the association between DM and tinnitus. The OR data and corresponding SE were derived from either the reported 95% CI or *P* values. These values were then logarithmically transformed to stabilize variance and normalize the distribution [29]. Statistical heterogeneity was assessed using the *I*^2^ statistic, where values of 25%, 50%, and 75% indicate low, moderate, and high heterogeneity, respectively [32]. Given the variability in the included studies—such as differences in population characteristics, diabetes type, and tinnitus diagnosis methods—significant clinical heterogeneity was identified. Therefore, the meta-analysis was conducted using the inverse variance (IV) method with a random-effects model to account for potential heterogeneity [29]. Sensitivity analyses were performed by excluding one study at a time, as well as by removing studies with lower NOS scores (<6) and those contributing to heterogeneity [33]. Subgroup analyses were conducted to explore the influence of study characteristics, including geographical region, mean age, sex distribution, tinnitus diagnosis methods, tinnitus prevalence, and the analytical model used (univariate or multivariate). The medians of continuous variables were used as cutoff values to define subgroups. Potential publication bias was evaluated using funnel plots, and Egger’s regression test was employed to detect small-study effects [34]. A *P* value of less than 0.05 in Egger’s test was considered indicative of publication bias. Statistical analyses were performed using RevMan (Version 5.1; Cochrane Collaboration, Oxford, UK) and Stata software (Version 17.0; Stata Corporation, College Station, TX).

## Results

### Literature search and study identification

**Figure 1.**
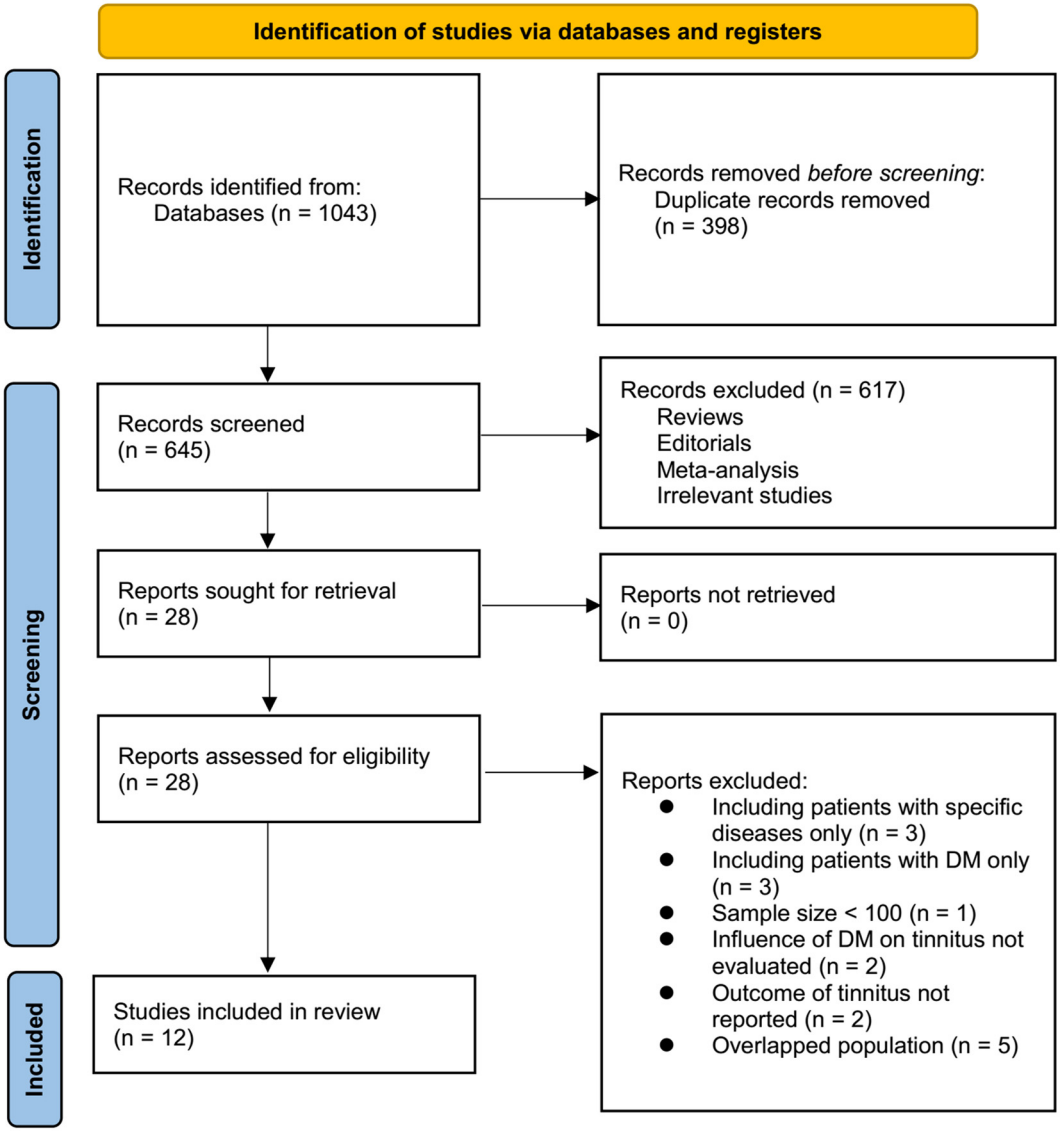
**PRISMA flowchart of study inclusion.** DM: Diabetes mellitus.

The initial search across the three databases yielded 1043 records. After duplicate removal, 645 unique articles remained. Title and abstract screening narrowed these down to 28 studies for full-text review. Ultimately, 12 studies met the inclusion criteria, encompassing a total of 2,277,719 participants aged 16–27. A detailed PRISMA flow diagram is shown in Figure 1.

### Study characteristics

The characteristics of the included studies are summarized in Table 1. These studies were published between 2015 and 2024 and conducted in Italy, China, the United States, Taiwan, the Netherlands, Brazil, and Korea. The study designs included two case-control studies [16, 26] and ten cross-sectional studies [17–25, 27]. Seven studies included participants from the general community population [20–22, 24–27], two studies involved individuals visiting Otolaryngology or Audiology Clinics [16, 18], and three studies included participants undergoing health check-ups [17, 19, 23]. The mean ages of the participants ranged from 38.4 to 72.1 years, and the proportions of men ranged from 0% to 64.2%. The diagnosis of DM was confirmed by medical history in 11 studies [16–24, 26, 27] and by International Classification of Disease (ICD) codes in one study [25]. Most studies assessed the influence of overall DM (type 1 and/or type 2 DM) [16, 17, 19–27], while one study specifically evaluated the influence of type 2 DM (T2DM) on the prevalence of tinnitus [18]. Tinnitus validation methods varied across studies. Five studies used clinical diagnosis [16–19, 22], six studies relied on self-reported tinnitus symptoms [20, 21, 23, 24, 26, 27], and one study used ICD codes [25]. Overall, 552,247 participants (24.2%) were identified as having tinnitus. When examining the association between DM and tinnitus, univariate analyses were employed in four studies [20, 25–27], while multivariate analyses were used in the remaining eight studies [16–19, 21–24], adjusting for at least age and sex.

**Table 1:** Characteristics of the included studies

**Study**	**Location**	**Study design**	**Participant characteristics**	**Sample size**	**Mean age (years)**	**Men (%)**	**Methods for diagnosis of DM**	**Type of DM**	**No. of participants with DM**	**Methods for diagnosis of tinnitus**	**Number of patients with tinnitus**	**Adjusted/matched variables**
Martines, 2015	Italy	CC	Subjects visiting an audiology department	120	57.6	64.2	Medical history	T1DM or T2DM	14	Clinical diagnosis	46	Age and sex
Hong, 2016	China	CS	Participants of health check	1596	48.1	46.9	Medical history	T1DM or T2DM	NR	Clinical diagnosis	300	Age, sex, BMI, and comorbidities
Li, 2018	China	CS	Subjects visiting an otolaryngology department	149	55.1	56.1	Medical history	T2DM	51	Clinical diagnosis	58	Age, sex, and BMI
Staudt, 2019	USA	CS	Community population aged 20–59 years	2511	38.4	47.8	Medical history	T1DM or T2DM	125	Self-reported symptom of tinnitus	589	None
Chang, 2019	Taiwan	CS	Participants of health check aged 65 years or older	597	72.1	53.9	Medical history	T1DM or T2DM	123	Clinical diagnosis	191	Age and sex
Qian, 2020	USA	CS	Community population aged 20–69 years	2705	54.3	49.7	Medical history	T1DM or T2DM	510	Clinical diagnosis	499	Age, sex, hearing loss, noise exposure, and comorbidities
Loiselle, 2020	The Netherlands	CS	Community population aged 25– 49 years	72709	49.9	40.4	Medical history	T1DM or T2DM	NR	Self-reported symptom of tinnitus	2944	Age, sex, hearing loss, BMI, and comorbidities
Chamouton, 2021	Brazil	CS	People visiting a healthcare service	1569	59.2	41.7	Medical history	T1DM or T2DM	NR	Self-reported symptom of tinnitus	496	Age, sex, comorbidities, and concurrent medications
Choi, 2021	Korea	CS	Community population	12537	38.9	52.5	Medical history	T1DM or T2DM	486	Self-reported symptom of tinnitus	2221	Age, sex, comorbidities, and depressive symptoms
Kuang, 2022	Taiwan	CS	Community population aged 18 years or older	2170728	55.1	42.8	ICD codes	T1DM or T2DM	450648	ICD codes	542682	None
Zeleznik, 2023	USA	CC	Community women aged 30–55 years	6477	52	0	Medical history	T1DM or T2DM	975	Self-reported symptom of tinnitus	488	None
Lee, 2024	Korea	CS	Community population aged 60 years or older	6021	69.3	48.9	Medical history	T1DM or T2DM	1215	Self-reported symptom of tinnitus	1733	None

DM: Diabetes mellitus; T1DM: Type 1 diabetes mellitus; T2DM: Type 2 diabetes mellitus; CC: Case-control studies; CS: Cross-sectional studies; ICD: International Classification of Diseases; NR: Not reported; BMI: Body mass index.

### Risk of bias

Detailed study quality evaluation via the NOS score is shown in Table 2. The quality assessment using the NOS revealed that 11 studies scored 6 stars or more [16–24, 26, 27], indicating moderate to high quality. Most studies were rated favorably regarding selection criteria; however, comparability between groups and outcome assessment exhibited greater variability. Notably, some studies lacked adequate control for confounding variables, thereby increasing the potential for bias [20, 25–27]. Despite these limitations, all studies were included in the primary analysis.

**Table 2:** Study quality evaluation via the Newcastle-Ottawa scale

**Study**	**Adequate definition of cases**	**Representativeness of cases**	**Selection of controls**	**Definition of controls**	**Control for age and sex**	**Control for other confounders**	**Exposure ascertainment**	**Same methods for events ascertainment**	**Non-response rates**	**Total**
Martines, 2015	1	0	1	1	1	0	1	1	1	7
Hong, 2016	1	0	1	1	1	1	1	1	1	8
Li, 2018	1	0	1	1	1	1	1	1	1	8
Staudt, 2019	0	1	1	1	0	0	1	1	1	6
Chang, 2019	1	1	1	1	1	0	1	1	1	8
Qian, 2020	1	1	1	1	1	1	1	1	1	9
Loiselle, 2020	0	0	1	1	1	1	1	1	1	7
Chamouton, 2021	0	0	1	1	1	1	1	1	1	7
Choi, 2021	0	1	1	1	1	1	1	1	1	8
Kuang, 2022	0	1	1	1	0	0	0	1	1	5
Zeleznik, 2023	0	1	1	1	0	0	1	1	1	6
Lee, 2024	0	1	1	1	0	0	1	1	1	6

### Meta-analysis results

**Figure 2.**
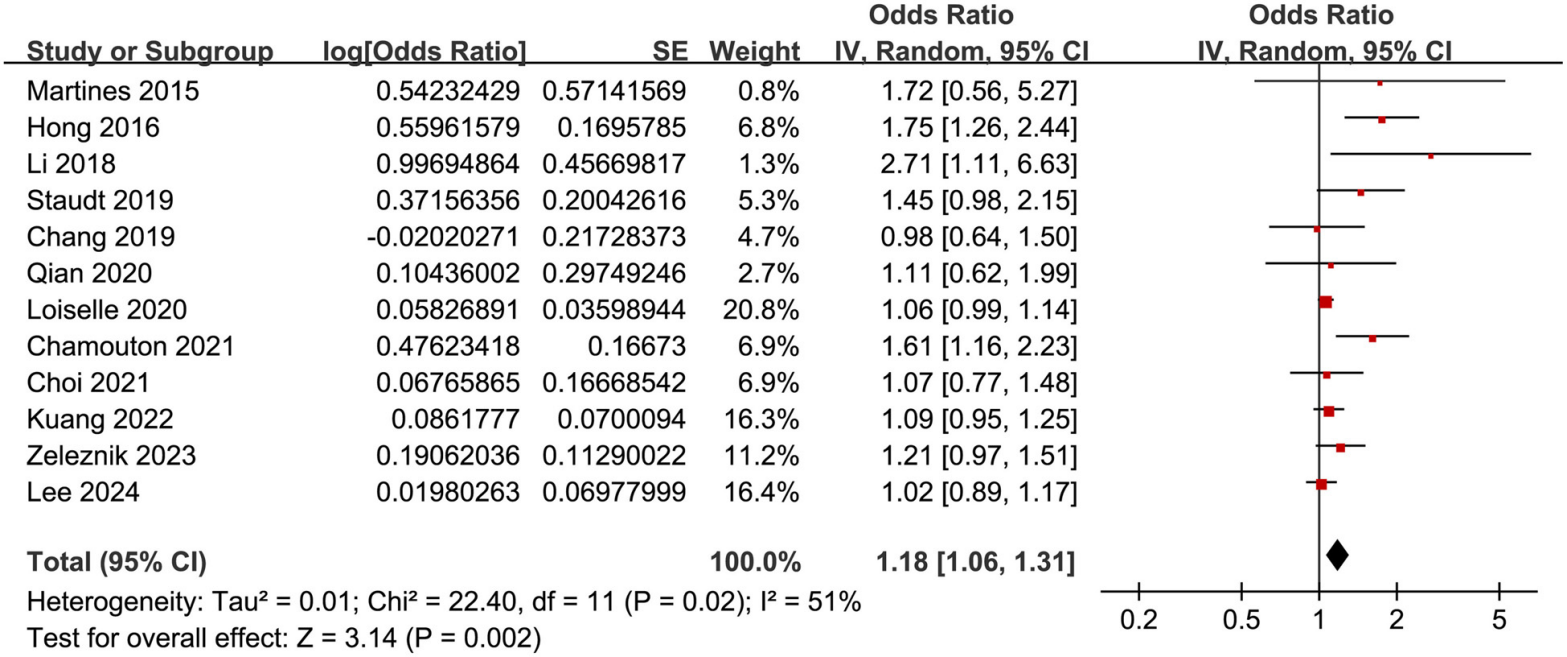
**Forest plots for the meta-analysis of the association between DM and tinnitus.** DM: Diabetes mellitus; CI: Confidence intervals.

**Figure 3.**
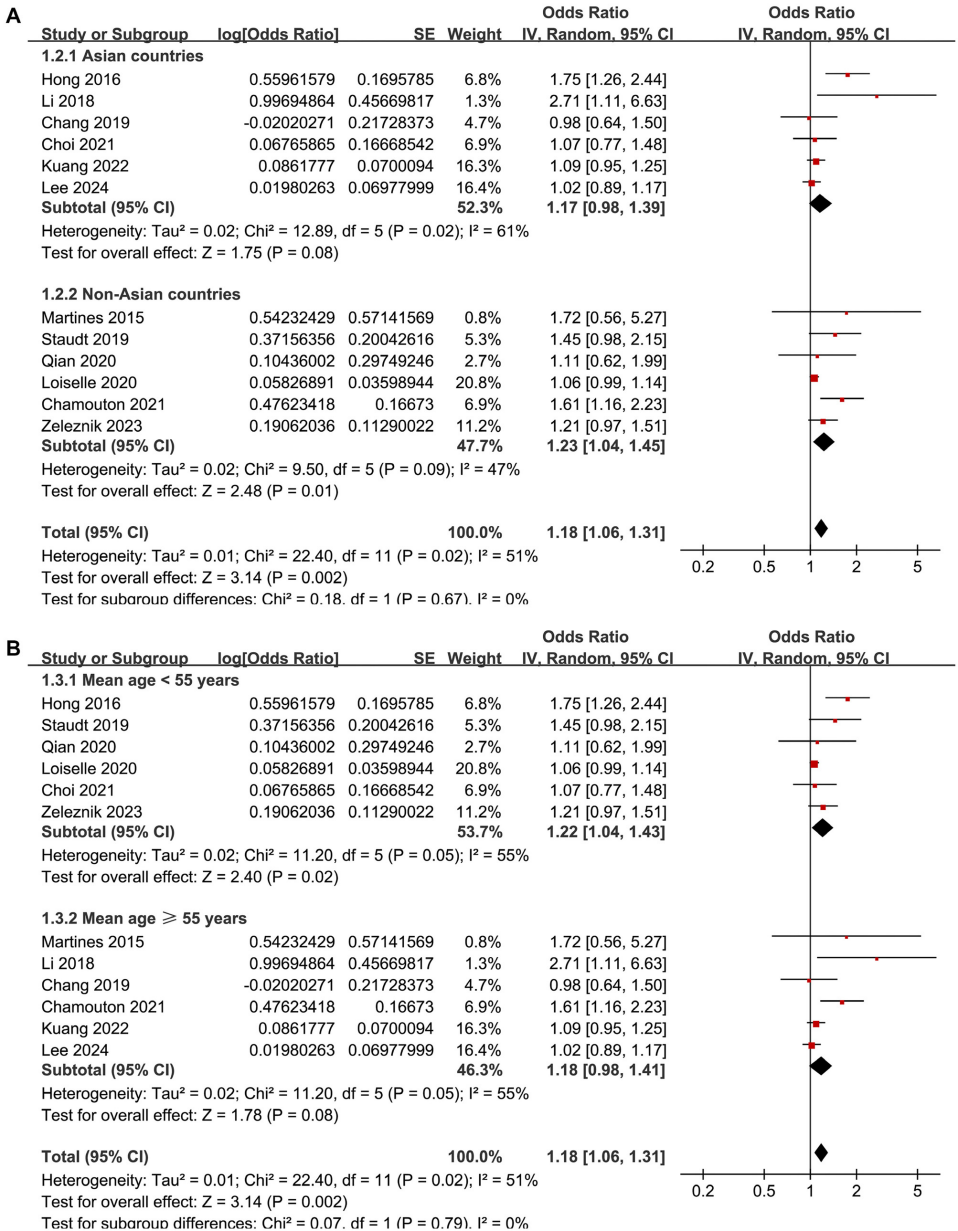
**Forest plots for the subgroup analyses of the association between DM and tinnitus.** (A) The subgroup analysis according to study country and (B) The subgroup analysis according to the mean age of the participants. DM: Diabetes mellitus; CI: Confidence intervals.

**Figure 4.**
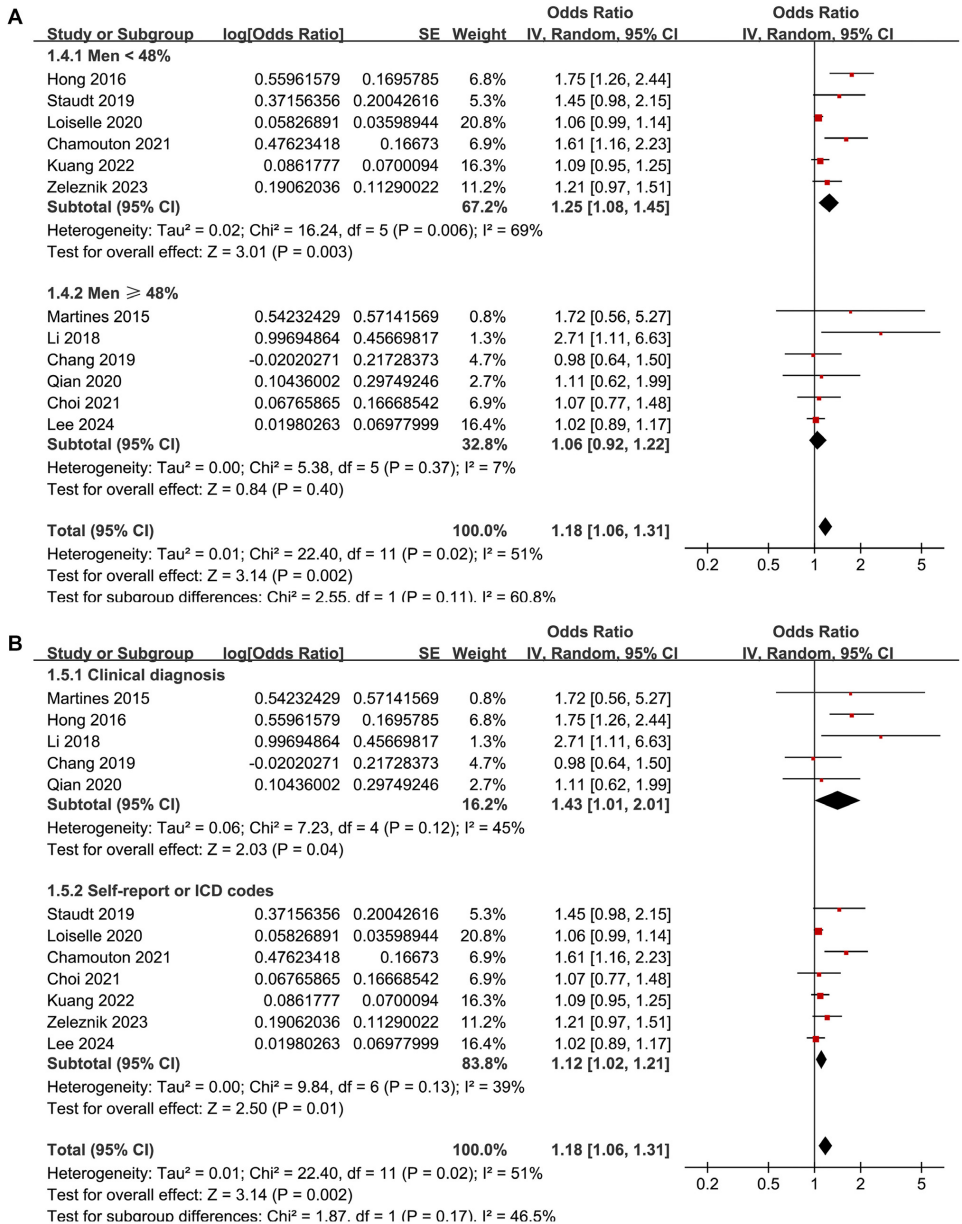
**Forest plots for the subgroup analyses of the association between DM and tinnitus.** (A) The subgroup analysis according to the proportion of men and (B) The subgroup analysis according to the methods for diagnosis of tinnitus. DM: Diabetes mellitus; CI: Confidence intervals.

**Figure 5.**
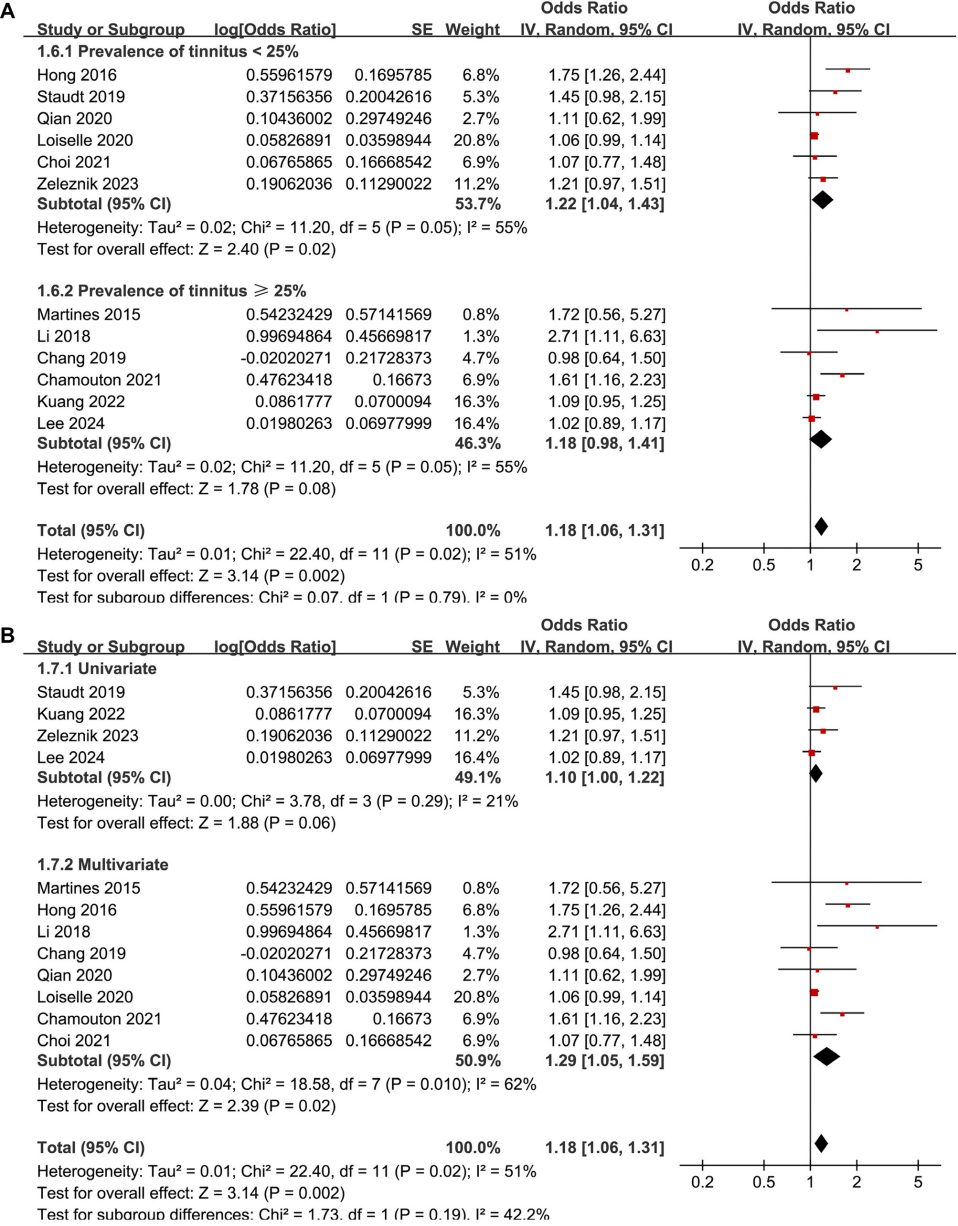
**Forest plots for the subgroup analyses of the association between DM and tinnitus.** (A) The subgroup analysis according to the prevalence of tinnitus in each study and (B) The subgroup analysis according to the analytic model for estimating the association between DM and tinnitus. DM: Diabetes mellitus; CI: Confidence intervals.

The meta-analysis demonstrated a significant association between DM and tinnitus (OR: 1.18, 95% CI: 1.06–1.31, *P* ═ 0.002; Figure 2). Moderate between-study heterogeneity was detected (*I*^2^ ═ 51%), indicating some variability in effect estimates. A sensitivity analysis, performed by excluding one study at a time, showed consistent results (OR: 1.12–1.23, *P* all < 0.05). Specifically, excluding the only study [18] that evaluated the influence of T2DM yielded similar findings (OR: 1.16, 95% CI: 1.05–1.27, *P* ═ 0.003; *I*^2^ ═ 46%). Similarly, excluding the single study with a NOS score of five also produced consistent results (OR: 1.21, 95% CI: 1.07–1.38, *P* ═ 0.003; *I*^2^ ═ 55%). These results suggest that the findings are robust and not overly influenced by the inclusion of lower-quality studies or outliers with extreme effect sizes. Subsequent subgroup analyses indicated that the association between DM and tinnitus was not significantly impacted by various study characteristics. These included the study location (*P* for subgroup difference ═ 0.67; Figure 3A), mean participant age (*P* ═ 0.79; Figure 3B), proportion of male participants (*P* ═ 0.11; Figure 4A), tinnitus diagnosis methods (*P* ═ 0.17; Figure 4B), tinnitus prevalence within studies (*P* ═ 0.79; Figure 5A), and the statistical models used to estimate the association (*P* ═ 0.19; Figure 5B).

### Publication bias

**Figure 6.**
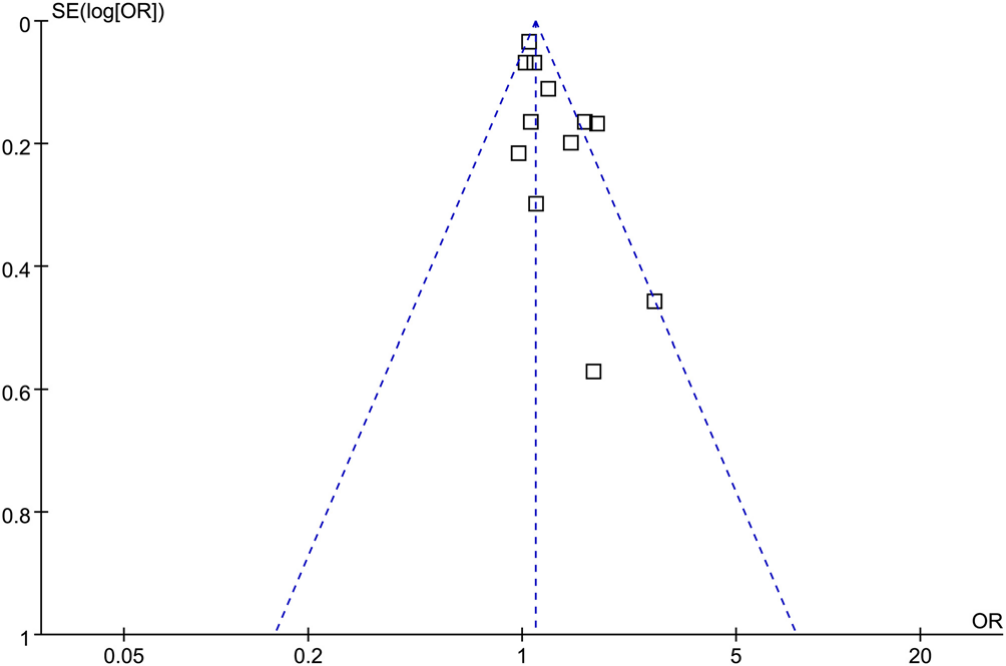
**Funnel plots for the publication bias underlying the meta-analysis of the association between DM and tinnitus.** DM: Diabetes mellitus.

The funnel plots for the meta-analysis examining the association between diabetes and tinnitus are presented in Figure 6. A visual assessment of the funnel plot indicated no notable asymmetry, a finding corroborated by Egger’s regression test, which showed no evidence of significant publication bias (*P* ═ 0.29). These results imply a low probability of small-study effects or selective reporting, thereby strengthening the reliability of the meta-analysis findings.

## Discussion

The findings of this meta-analysis suggest a significant association between DM and tinnitus, with a pooled OR of 1.18, indicating that individuals with DM have an 18% higher likelihood of experiencing tinnitus compared to non-diabetic individuals. This association remained consistent across various sensitivity analyses, which excluded lower-quality studies and those contributing to heterogeneity. The consistent results reinforce the robustness of the association and suggest that DM may indeed be a risk factor for tinnitus. Previous studies have reported a range of ORs in examining this relationship, but our meta-analysis provides a more comprehensive conclusion by encompassing data from multiple studies with a large number of participants. Several biological mechanisms may underlie the relationship between DM and tinnitus. DM is characterized by chronic hyperglycemia, which can lead to microvascular and macrovascular complications, including damage to the auditory pathways [35, 36]. The cochlea, which has a high metabolic demand, may be particularly vulnerable to DM-related vascular damage. Chronic hyperglycemia can reduce blood flow to the inner ear, leading to ischemic damage and contributing to tinnitus [37]. Tinnitus may also arise from neuroplastic changes in the dorsal cochlear nucleus (DCN), where hyperglycemia-induced oxidative stress and microvascular alterations may result in dysregulated neural activity [38]. These changes disrupt the balance of excitatory and inhibitory signaling, contributing to tinnitus perception [39]. Additionally, DM is a significant cause of peripheral neuropathy, which can impair auditory nerve function and result in abnormal firing patterns that the central auditory system interprets as tinnitus [8]. This highlights the role of DM-related nervous system damage in tinnitus, independent of cochlear issues [40]. Furthermore, DM can induce oxidative stress and inflammation, both of which are implicated in the pathogenesis of tinnitus [41, 42]. The glycation of proteins and lipids in DM leads to the formation of advanced glycation end products (AGEs), resulting in cellular dysfunction and inflammation in auditory tissues, further exacerbating tinnitus symptoms [43–45]. Given the complex interplay of these mechanisms, it is plausible that DM affects tinnitus through a combination of vascular, inflammatory, and metabolic pathways.

The sensitivity analyses conducted in this study were essential in confirming the robustness of the findings. Sequentially excluding individual studies did not lead to significant changes in the pooled effect estimates, demonstrating that the results were not disproportionately influenced by any single study. Furthermore, excluding the only study that specifically examined T2DM, as well as excluding lower-quality studies based on the NOS score, produced results consistent with the overall analysis. These findings suggest that the observed association between DM and tinnitus is neither restricted to a specific type of diabetes nor significantly affected by study quality. The moderate heterogeneity observed (*I*^2^ ═ 51%) was effectively addressed using a random-effects model, which accounts for variability among studies. Subgroup analyses were also performed to identify potential sources of heterogeneity. However, no significant differences were found based on factors, such as geographical region, mean age, sex distribution, tinnitus diagnostic methods, or statistical models employed. This consistency across subgroups suggests that the association between DM and tinnitus may be generalizable to diverse populations and clinical settings. While this meta-analysis has several strengths, certain limitations should be acknowledged. First, the observational nature of the included studies limits the ability to infer causality between DM and tinnitus. Although the analysis provides evidence of an association, further prospective longitudinal studies are necessary to determine the temporal relationship and investigate whether DM directly contributes to tinnitus or whether other factors mediate this relationship. Second, there was variation in how DM and tinnitus were diagnosed across studies, introducing the possibility of misclassification bias. For example, some studies relied on self-reported tinnitus, whereas others used clinical diagnoses or ICD codes, which may differ in accuracy. Similarly, the methods for diagnosing DM ranged from medical records to ICD codes, with potential inconsistencies in diagnostic criteria influencing the observed association. Lastly, while most studies adjusted for key confounders, such as age and sex, residual confounding from unmeasured factors—such as comorbid conditions, lifestyle factors, or medication use—may still affect the association between DM and tinnitus.

The strengths of this meta-analysis include a comprehensive search strategy spanning multiple databases, the use of an established quality assessment tool NOS, and a thorough examination of heterogeneity through sensitivity and subgroup analyses. The large sample size and inclusion of studies from diverse geographic regions enhance the generalizability of the findings. By incorporating studies with a minimum sample size of 100 participants and excluding populations with specific diseases (e.g., cardiovascular or neurological disorders), the analysis minimizes potential confounding factors and strengthens the reliability of its conclusions. Although the 100-participant cutoff is somewhat arbitrary, this criterion was implemented to mitigate the influence of small, underpowered studies, which can lead to unstable results or exaggerated effect sizes [46]. Smaller studies are more susceptible to confounding and random error, which can bias meta-analytic findings [30]. Prior research has emphasized the risks of including underpowered studies in meta-analyses, as they often lack sufficient statistical power to detect clinically meaningful differences [30]. Furthermore, the use of a random-effects model accounts for between-study variability, bolstering the validity of the pooled results. The clinical implications of these findings are significant, underscoring the need for heightened awareness among healthcare providers about the potential association between DM and tinnitus. Patients with DM should be monitored for auditory symptoms, as early identification of tinnitus in this population could prompt timely interventions to manage both conditions more effectively [47, 48]. Tinnitus, which can adversely impact quality of life by contributing to hearing difficulties, sleep disturbances, and psychological stress, should be recognized as an important comorbidity in individuals with DM. In particular, those with DM should prioritize sleep quality and duration, as poor sleep has been associated with elevated levels of fibroblast growth factor (FGF), a factor that can exacerbate diabetic complications [49]. The interplay between sleep disturbances and diabetes-related complications is mediated by mechanisms, such as increased oxidative stress, dysregulation of appetite-regulating hormones, and heightened sympathetic nervous system activity [50]. By highlighting these interconnections, the findings provide a deeper understanding of how managing sleep may alleviate tinnitus symptoms and improve overall metabolic health in individuals with DM. Additionally, the meta-analysis suggests that controlling blood glucose levels and addressing DM-related complications could reduce the risk or severity of tinnitus. Nonetheless, further research is needed to confirm these hypotheses and translate them into clinical practice.

Future research should prioritize prospective cohort studies to investigate the temporal relationship between DM and tinnitus, with a strong focus on controlling for potential confounders, such as age, sex, lifestyle factors, and comorbid conditions. Additionally, studies that delve into the pathophysiological mechanisms connecting DM and tinnitus are necessary to illuminate the causal pathways involved. Exploring the roles of different types of DM, particularly the distinct contributions of type 1 and type 2 diabetes, could further clarify these mechanisms. Furthermore, evaluating interventions aimed at improving glycemic control and mitigating DM-related complications may reveal their potential to prevent or reduce tinnitus in diabetic patients [36]. Lastly, the development of standardized diagnostic criteria for both DM and tinnitus in future studies would enhance result comparability and strengthen the evidence base for this association.

## Conclusion

In conclusion, this meta-analysis demonstrates a significant association between DM and tinnitus, revealing an 18% increased likelihood of tinnitus in individuals with DM. The consistency of results across sensitivity and subgroup analyses highlights the robustness of these findings. Although the precise mechanisms underlying this association are not yet fully understood, factors, such as vascular damage, inflammation, oxidative stress, and metabolic dysregulation are likely contributors to the development of tinnitus in individuals with DM. These findings carry potential clinical implications for the management of patients with DM. However, further research is needed to establish a causal relationship and investigate potential interventions to reduce the risk of tinnitus in this population.

## Supplemental data

**PubMed (*n* ═ 249)**

(“diabetes mellitus”[MeSH] OR “diabetes” OR “diabetic” OR “type 2 diabetes” OR “T2D” OR “T2DM” OR “type 1 diabetes” OR “T1D” OR “T1DM”) AND (“tinnitus” [MeSH] OR “tinnitus”)

**Embase (*n* ═ 562)**

(“diabetes mellitus”/exp OR “diabetes”“ OR “diabetic” OR “type 2 diabetes” OR “T2D” OR “T2DM” OR “type 1 diabetes” OR “T1D” OR “T1DM”) AND (“tinnitus”/exp OR “tinnitus”)

**Web of Science (*n* ═ 232)**

TS= (“diabetic” OR “diabetes” OR “diabetes mellitus” OR “type 2 diabetes” OR “T2D” OR “T2DM” OR “type 1 diabetes” OR “T1D” OR “T1DM”) AND TS ═ (“tinnitus”)

## Data Availability

All the data generated during the study was within the manuscript and the supplemental materials.
